# Different Modes of Low-Frequency Focused Ultrasound-Mediated Attenuation of Epilepsy Based on the Topological Theory

**DOI:** 10.3390/mi12081001

**Published:** 2021-08-23

**Authors:** Minjian Zhang, Bo Li, Yafei Liu, Rongyu Tang, Yiran Lang, Qiang Huang, Jiping He

**Affiliations:** 1 School of Mechatronical Engineering, Beijing Institute of Technology, Beijing 100081, China; 3120170166@bit.edu.cn (M.Z.); 3120170125@bit.edu.cn (B.L.); yafei.liu@bit.edu.cn (Y.L.); qhuang@bit.edu.cn (Q.H.); 2 Beijing Advanced Innovation Center for Intelligent Robots and Systems, Beijing Institute of Technology, Beijing 100081, China; tangrongyu@semi.ac.cn (R.T.); yiran.lang@bit.edu.cn (Y.L.)

**Keywords:** epilepsy, ultrasound, pulsed wave, continuous wave, EEG, brain connections

## Abstract

Epilepsy is common brain dysfunction, where abnormal synchronized activities can be observed across multiple brain regions. Low-frequency focused pulsed ultrasound has been proven to modulate the epileptic brain network. In this study, we used two modes of low-intensity focused ultrasound (pulsed-wave and continuous-wave) to sonicate the brains of KA-induced epileptic rats, analyzed the EEG functional brain connections to explore their respective effect on the epileptic brain network, and discuss the mechanism of ultrasound neuromodulation. By comparing the brain network characteristics before and after sonication, we found that two modes of ultrasound both significantly affected the functional brain network, especially in the low-frequency band below 12 Hz. After two modes of sonication, the power spectral density of the EEG signals and the connection strength of the brain network were significantly reduced, but there was no significant difference between the two modes. Our results indicated that the ultrasound neuromodulation could effectively regulate the epileptic brain connections. The ultrasound-mediated attenuation of epilepsy was independent of modes of ultrasound.

## 1. Introduction

Epilepsy is a common neurological disorder characterized by recurrent seizures and caused by abnormal, highly synchronized discharges from a plurality of neurons [1,2]. Recurrent seizures could be associated with comorbidities such as cognitive deficits, mood and anxiety disorder, traumas and sudden death in epilepsy [3,4,5]. Epilepsy affects more than 50 million people worldwide, with an annual cumulative incidence of 68 per 100,000 persons [1,6,7]. Developing countries suffer a higher incidence rate than developed ones [6,7]. While over 20 mediations have been developed for the management of epilepsy, over one-third of epilepsy patients do not respond to any medication [1,8]. For drug-resistant epilepsy (DRE) patients with focal or regional onset, surgery on epileptic lesions and deep brain stimulation have palliative effects. These invasive surgical methods are inevitably accompanied by unpredictable risks [9,10,11]. In recent years, the ketogenic diet has been increasingly used to treat epilepsy refractory to antiepileptic drugs and other neurological disorders [12,13]. The ketogenic diet is a high-fat, low-carbohydrate, and normal-protein diet that has gradually become an effective tool to control antiseizure drug-refractory epilepsy [14]. Nevertheless, in approximately 25% of individuals with epilepsy, seizures cannot be controlled by any available treatment. Therefore, improved noninvasive curative therapeutic approaches for epilepsy treatment are urgently needed. To meet this demand, we began studying the impact of ultrasound neuromodulation on epilepsy. In the present study, we investigated the effect of two modes of ultrasound on the brain functional connectivity of epileptic rats.

Low-frequency ultrasound has lately received great attention as an effective method for transcranial neuromodulation due to its high spatial resolution, noninvasiveness, and safe characteristics [15,16]. Ultrasound can be transmitted as continuous or pulsed waves through tissues, including bone, and can be thermal and/or nonthermal (mechanical) [17,18,19]. Currently, pulsed waves are the most commonly used mode in ultrasound neuromodulation. Tufail et al. discovered that transcranial pulsed ultrasound could directly stimulate intact brain circuit activity in mice [20]. Transcranial pulsed ultrasound can modulate neuronal activity [20,21], neural network connections [22], and cerebral hemodynamics [23,24,25]. Pulsed ultrasound has been shown to reduce the occurrence of EEG bursts and the severity of epileptic comorbidities [26]. Hakimova et al. found that pulsed ultrasound could also inhibit spontaneous recurrent seizures in the acute period of epilepsy and improve the performance of behavioral tests evaluating depression and sociability during chronic epilepsy [27]. Pulsed ultrasound has also been proven to modulate the nonlinear dynamics of local field potentials in temporal lobe epilepsy of mice [28]. King et al. reported that short-duration continuous-wave ultrasound stimuli were equally, if not more, effective than pulsed stimuli in eliciting motor responses [29]. Moreover, Li and colleagues found that continuous and pulsed waves decreased the intensity of the power spectrum in local field potentials of epileptic mice and increased the interval between seizures [30].

The brain is a complex network composed of interacting subsystems, and it is now generally accepted that synchronization has a crucial role in brain functioning and dysfunction [31,32,33,34,35]. A salient example of pathophysiological neuronal synchronization is epilepsy and its cardinal symptom, seizures. The construction of the brain function network can reflect well the activity state of the whole brain. Common indicators of the brain network, clustering coefficient, path length, etc., are often used to assess the level of whole-brain activity in a certain state and to explore brain mechanisms. Epilepsy is a brain pathology that is closely related to neural synchronization phenomena [36]. According to a number of previous studies, the epileptic focus is the region where epilepsy begins that serves as the target of surgical intervention [37]. From the perspective of the epileptic circuit, many brain regions have important roles in the initial stage of epilepsy. The formation of a single epileptic focus does not necessarily cause a seizure. The epileptic circuit is the physical and physiological basis of seizures [38]. Anatomical and clinical observations, as well as invasive EEG and functional neuroimaging, have supplied increasing evidence for the existence of specific cortical and subcortical epileptic networks in the occurrence and expression of not only primary generalized but also focal onset seizures [39,40,41,42,43,44,45,46], thus suggesting that the seizures might be a more complex network, where different brain regions have different roles in the development of epilepsy. It is foreseeable that further developments of synchronization and topological theory, as well as refined modeling approaches, will further deepen our understanding of synchronization phenomena underlying the generation and termination of seizures in epileptic brain networks [36]. Yet, related experiments on different modes of ultrasound neuromodulation and its influence on epileptic brain networks are still missing.

Previous reports have confirmed that ultrasound (US) has an inhibitory effect on epilepsy. In previous studies, we explored the regulation of low-intensity transcranial pulsed ultrasound (LITPU) on epilepsy [47]. In this study, we used two different modes of ultrasound on kainic acid (KA)-induced epileptic rats in order to uncover the influence of different modes of ultrasound on the epileptic brain network. KA is a potent neuroexcitatory and neurotoxic analog of glutamate. The intraperitoneal administration of KA can induce tonic-clonic seizure and limbic motor signs in rats, including wet dog shakes, facial myoclonia, and paw tremor [48]. The seizures are on account of neuron damage, especially in the hippocampus and amygdaloid complex [49]. The present study provided further guidance for applying ultrasound neuromodulation in the treatment of epilepsy and provided a new way of thinking for exploring multi-mode and multi-parameter ultrasound neuromodulation. This may lead to new developments for diagnosis, control, and treatment of the neurological disease epilepsy.

## 2. Materials and Methods

### 2.1. Generation and Characterization of Ultrasound Waveforms

**Figure 1:**
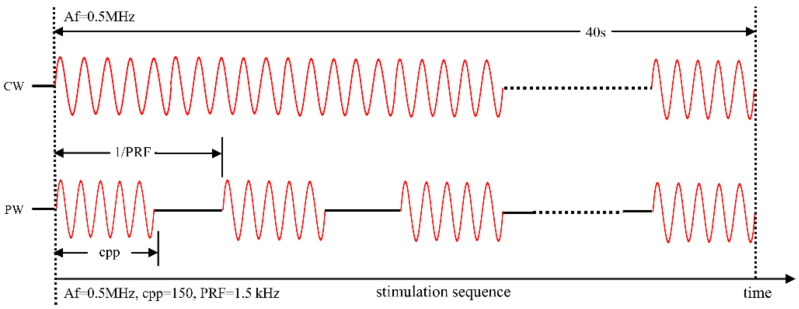
Ultrasound parameters. Pulsed ultrasound waveform (pw) and continuous ultrasound waveform (cw) generation with the following properties: Af = 0.5 MHz, c.p.p (cycles per pulse) = 150, PRF (pulse-repetition frequency) = 1.5 kHz; continuous ultrasound waveform generation with the following properties: Af = 0.5 MHz.

**Figure 2:**
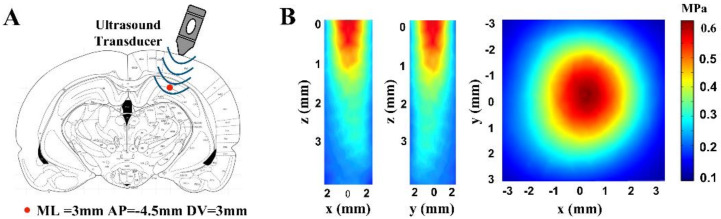
Ultrasound stimulation region and ultrasound distribution. (**A**) The region for ultrasound stimulation (ML = 3 mm, AP = −4.5 mm, DV = 3 mm relative to Bregma). (**B**) The two-dimensional sonication distribution in the xz, yz, and xy planes.

Optimal waveforms between transcranial transmission and brain absorption for evoking intact brain circuit activity have been reported to be composed of acoustic frequencies ranging from 0.25 to 0.65 MHz. We used immersion-type US transducers with a 0.5 MHz center frequency (35 mm focal depth, 20 mm in diameter; Goworld, Guangdong, China) to produce ultrasound waveforms. For the pulsed wave (pw), pulses were generated by a two-channel waveform generator (33500B, Keysight Technologies Inc., Santa Rosa, CA, USA) and amplified through a radio-frequency amplifier (North Star model SWA200D RF power amplifier, The Institute of Acoustics of the Chinese Academy of Sciences, Beijing, China). As shown in Figure 1, the Af, c.p.p, and PRF of pulsed ultrasound were 0.5 MHz, 150, and 1.5 kHz, respectively. Continuous waves (cw) were generated by one channel of the generator. The Af of the continuous ultrasound was 0.5 MHz. The ultrasound stimulation time of these two modes was 40 s. A study on the ultrasound neuromodulation experimental platform was published at the ICCIIBMS conference [50]. The ultrasound transducer was fixed over the rat and linked to the rat skull through a 3D-printed conical acoustic collimator filled with US gel. A calibrated needle-type hydrophone (PT-1711395, The Institute of Acoustics of the Chinese Academy of Sciences, Beijing, China) was used to measure the two-dimensional ultrasound distribution in the xz, yz, and xy planes. The maximum ultrasound pressure of both pulsed and continuous ultrasound was 0.657 MPa. The ultrasound stimulation region and ultrasound distribution are shown in Figure 2.

### 2.2. Animal Grouping and EEG Setup

A total of 27 Sprague-Dawley rats (all male, body weight 300 ± 50 g, Beijing SPF Bio-technology Co., Ltd., Beijing, China) were housed in an environment with a temperature of 22 ± 1 °C, relative humidity of 50 ± 1%, and a light/dark cycle of 12/12 h and were given ad libitum access to food and water. Animal care and handling were in accordance with the guidelines approved by the Institutional Animal Care and Use Committee of the Beijing Institute of Technology. According to the current protocols approved by the Laboratory Animal Ethics Committee of Beijing Institute of Technology (Beijing, China), the experimental procedures in this study were aimed at minimizing the pain or discomfort of the animals.

Twenty-four rats were randomly divided into the three following groups: Pw Group (*n* = 8) that was treated with low-intensity pulsed ultrasound stimulation (LIPUS) after intraperitoneal injection of KA, recorded as ‘KA (+)/LIPUS (+)’; Cw Group (*n* = 8) that was treated with low-intensity continuous ultrasound stimulation (LICUS) after intraperitoneal injection of KA, recorded as ‘KA (+)/LICUS (+)’; Control Group (*n* = 8) that only received KA without sonication, recorded as ‘KA (+)/US (-).’ The rats in the control group (*n* = 8) only received sonication without epileptic induction, recorded as ‘KA (−)/US (+).’ Body temperature was maintained at ~37 °C using a temperature controller (serial no. C4L02-010, RWD Life Science Co., Ltd., Shenzhen, China) during all experiments. Two rats received LIPUS and LICUS, respectively, and one rat received no operations. These three rats were used for histological analysis.

**Figure 3:**
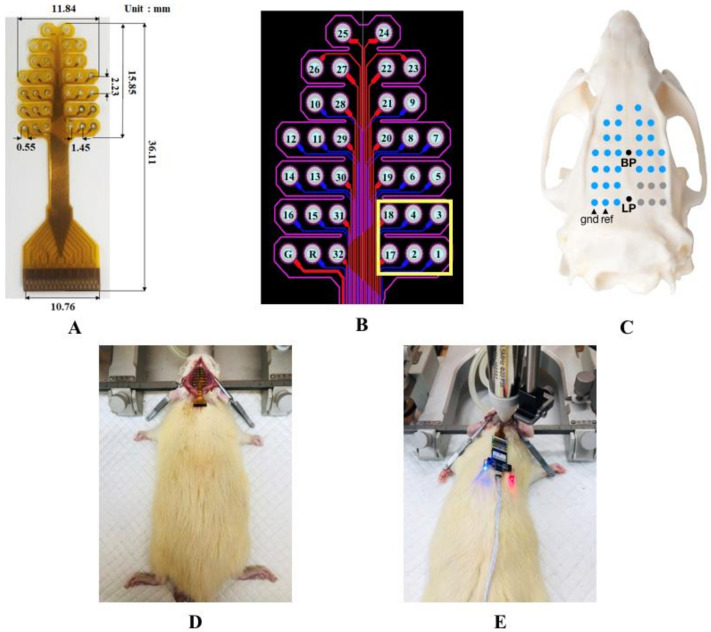
(**A**) Self-developed 32-channel EEG electrode and its size. (**B**) The electrode circuit diagram; the yellow frame is the position reserved for the ultrasound transducer, shown inside the yellow frame. (**C**) The relative position of the electrode on the skull surface. (**D**) Implantation with the EEG electrode. (**E**) A rat undergoing ultrasound neuromodulation.

One self-developed 32-channel EEG electrode was used to acquire EEG signals. Previous studies have reported on the effectiveness and safety of electrode use [47,51]. The size of the electrode is shown in Figure 3A. To have enough space to place the ultrasound transducer on the skull and avoid the chaos effect caused by the superposition of ultrasound and EEG, we removed the 1st, 2nd, 3rd, 4th, 11th, 17th, and 18 channels artificially, as shown in Figure 3B. The electrode array was fixed on rat skulls with cranial nails, as shown in Figure 3C,D. All surgical procedures were performed under full general anesthesia with 1.5% isoflurane in oxygen-enriched air using aseptic techniques. A gas evacuation apparatus (R546W, RWD Life Science Co., Ltd., Shenzhen, China) was used to maintain anesthesia throughout the procedure without interruption. The nails penetrated the rat skulls into the epidural space without damaging the dura mater. In comparison to craniotomy, this method greatly reduced the damage to animals, thus improving the stability and safety. After the electrode was fixed, a special adapter was used to connect the electrode to the EEG acquisition equipment (Plexon OmniPlex, Hong Kong Plexon Co., Ltd., Hong Kong, China).

### 2.3. Epileptic Induction and In Vivo Ultrasound Neuromodulation

**Figure 4:**
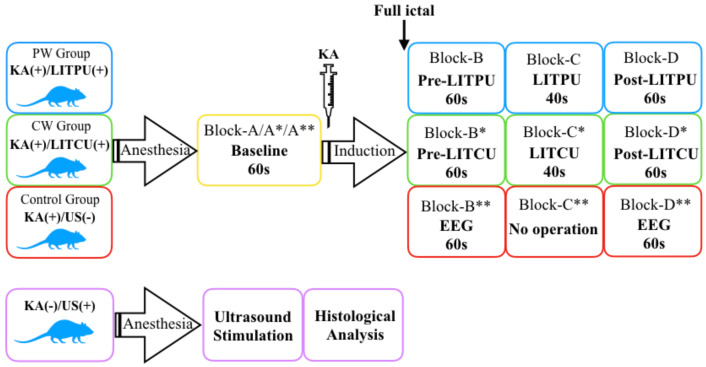
Experimental flow chart. * represents the cw group and ** represents the pw group.

EEG signals were recorded at a 2000 Hz sampling rate after the experiment started. Baseline data were recorded for 60 s (shown as ‘Block-A/A*/A**’ in Figure 4) in all three groups. The KA solution (6.5 mg/kg, based on animal weight, 2 mg/1 mL in 0.9% saline, No. k0250-10MG, Sigma-Aldrich, St Louis, MO, USA) was intraperitoneally injected into the rats in the pw group and the cw group. We evaluated the seizures of each rat by forelimb clonus and tail-twitches. After the epilepsy behavior could be clearly observed in the rats, EEG data were continuously recorded in the control group. Seizures were defined as EEG segments with continuous synchronous high-frequency and high-amplitude oscillations with a minimum duration of 10 s, and an amplitude at least twice that of the previous baseline [48,52]. After the epileptic EEG was recorded for 60 s, sonication was then applied according to preset parameters in the cw group and the pw group (noted as ‘Block-C’ and ‘Block-C*,’ respectively). After sonication, EEG data were recorded for the 60 s (noted as ‘Block-D’ and ‘Block-D*,’ respectively). Figure 4 shows all the experimental procedures of each group. When all operations were completed, the seizures were terminated by intraperitoneal injection of diazepam (10 mg/kg). The rats used for histological analysis only received sonication without epileptic induction. After all experimental processes were completed, the rats were euthanized using intraperitoneal injections of pentobarbital (150 mg/kg).

### 2.4. Data Acquisition and Processing

**Figure 5:**
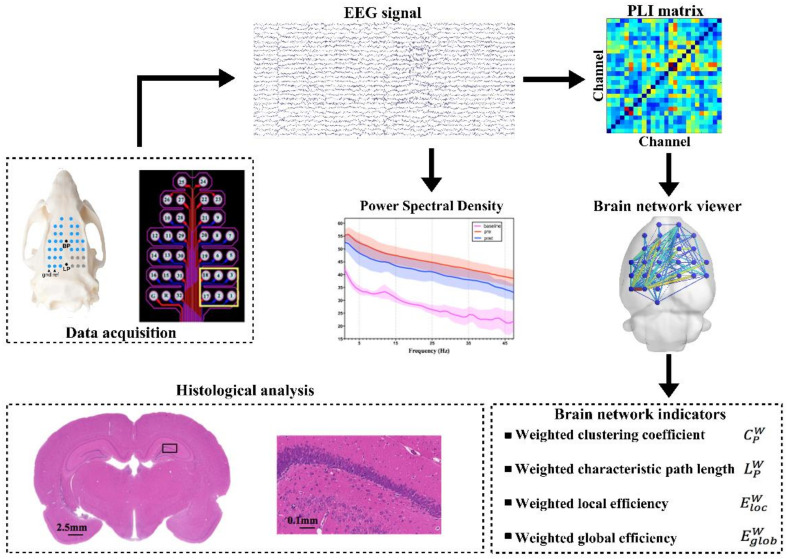
Flowchart for EEG signals processing and weighted functional connectivity.

The EEG signals were acquired by a Plexon OmniPlex at a 2000 Hz sampling rate, and electrode impedances were kept below 5 kΩ. The EEG data were further processed with the EEGLAB toolbox (Swartz Center for Computational Neuroscience, La Jolla, CA, USA). First, the EEG data were filtered (bandpass = 0.1–48 Hz) using a Basic FIR filter. Electrical interference from the 50 Hz-line noise and the ‘baseline drift’ was removed. After re-referencing, the EEGLAB function ‘runica’ was applied to analyze the processed data to obtain independent component (IC) sources. Regardless of the visual inspection of each IC scalp projection and power spectrum, some IC sources related to no-brain artifacts (eye movements, shaking head, etc.) were identified and removed to obtain clean EEG data. To better explore the differences in effective brain connectivity, delta (0.1~4 Hz), theta (4~8 Hz), alpha (8~12 Hz), beta (12~30 Hz), and gamma (30~48 Hz) bands were extracted in sequence from the pre-processed signals. Figure 5 shows a schematic of the EEG data processing flow.

### 2.5. Phase Lag Index Analysis and Construction of the Brain Connectivity Matrix

Some effective methods of brain functional connectivity for quantifying phase synchronization in multichannel EEG have been previously developed, such as imaginary components of coherency (IC) and phase coherence (PC). The *PLI* (phase lag index) can be used to acquire credible estimated values of phase synchronization, which are invariant to the existence of usual noise sources [53]. Considering two given EEG signals *x* and *y*, the PLI value is calculated by the following equations, where *k* = 1…N.

(1) $$PLI_{xy}=\left|<sign\left[\theta_{x}\left(t_{k}\right)-\theta_{y}\left(t_{k}\right)\right]>\right|$$

The interaction between two neural sources causes a coherent phase relationship between their corresponding time series at a value different from 0 and π. The PLI value ranges between 0 and 1, 0≤PLI≤1, where 0 indicates either no coupling or coupling with a phase difference centered around 0 mod π, and 1 indicates completed phase locking at a value of $\Delta \theta \left(t_{k}\right)=\theta_{x}\left(t_{k}\right)-\theta_{y}\left(t_{k}\right)$ different from 0 mod π. In Equation (1), $\left|x\right|$ denotes the absolute value of X, and <Y> denotes the mean of the vector, where ***sign*** () is a signum function, and the result of ***sign*** [Z_1_–Z_2_] denotes positive or negative 1; if Z_1_ > Z_2_, the value is 1; otherwise, the value is 0.

(2) $$\theta_{x}\left(t_{k}\right)=arctan\frac{\tilde{x}\left(t_{k}\right)}{x\left(t_{k}\right)}$$

(3) $$z\left(t_{k}\right)=x\left(t_{k}\right)+i\tilde{x}\left(t_{k}\right)=A\left(t_{k}\right)e^{i\theta \left(t_{k}\right)}$$

In (1), $\theta \left(t_{k}\right)$ can be calculated by (2), which represents the instantaneous phase of the signal. The analytical signal $z\left(t_{k}\right)$ is a complex value, $x\left(t_{k}\right)$ a real-time series, and $\tilde{x}\left(t_{k}\right)$ is its corresponding Hilbert transform.

Through the above calculation principles, the Hermes toolbox (Centre for Biomedical Technology, Technical University of Madrid, Madrid, Spain) was used to calculate the adjacency matrix formed by the functional brain connectivity, based on its built-in phase lag index algorithm [54]. For each subject, resting-state functional connectivity was captured by asymmetric matrices 26×26 symmetric *C_ij_*.

(4) $$C_{ij}=\left[\begin{array}{ccc} C_{11} & \cdots & C_{1n}\\ \vdots & \ddots & \vdots \\ C_{n1} & \cdots & C_{nn} \end{array}\right]$$

In this matrix, each node represents a channel in the electrode, each row and column corresponds to a different node, and the element of the matrix located at the junction of the *i*th and *j*th columns encode information about the connection between channels *i* and *j*. Connectivity matrix measurement was performed in the Brain Connectivity Toolbox [55]. The BrainNet Viewer toolbox was used to visualize the brain functional connectivity [56]. As a statistical feature of the functional brain connections, we calculated brain network indicators, including the characteristic path length Lp, clustering coefficient Eg, local efficiency Eg, and global efficiency Eloc. The abbreviations and descriptions of each indicators are shown in Table 1. In order to reduce the complexity of computation and make the definition of network metrics more clarified, most previous research have used binary networks to construct brain networks. However, the strength of connections below the threshold is ignored in binary networks, and details of the network structure cannot be identified, so the weighted networks were used for analysis in this study.

### 2.6. Histological Analysis

Histological observation of the rat brain regions treated with sonication was performed. To prepare brain tissue slices for histological observation, rats were deeply anesthetized by isoflurane, and transcardiac perfusion was carried out using 4% paraformaldehyde in PBS. Rat brains were then removed and fixed in 10% neutralized formalin at 4 °C overnight and dehydrated with 70%, 80%, 95%, and 100% gradient alcohol for 30 min each. In order to prepare the histological slices, the tissue was cut in the plane perpendicular to the ultrasonic path. Hematoxylin and eosin (H&E) were used to evaluate potential hemorrhaging and microscopic tissue injuries. For H&E staining, coronal slices were deparaffinized with xylene and rehydrated with 100%, 95%, and 80% ethanol. The slices were stained with hematoxylin, washed with distilled water, differentiated in 1% hydrochloric acid alcohol, washed with 1% ammonia solution, and stained with eosin. Under microscope observation, the nuclei were expected to be blue, and the cytoplasm to be pink. An inverted microscope (CI-S, Nikon Corporation Co., Ltd., Tokyo, Japan) was used for observation. An automatic digital pathology section scanner (KF-PRO-120, KFBIO technology for health Co., Ltd., Yuyao, China) was used for scanning sections.

### 2.7. Statistical Analysis

GraphPad Prism 8 software (GraphPad Software Inc., San Diego, CA, USA) was used for statistical analyses. In intergroup analysis, a one-way analysis of variance (ANOVA) was used to measure group differences in various brain network indicators. For intragroup analysis, one-way repeated measures ANOVA and the Geisser–Greenhouse correction were used to correct for nonsphericity [57]. Bonferroni correction was used to correct for the effect of multiple comparisons in neural oscillations. *p*-Value < 0.05, 0.01, 0.001, or 0.0001 was considered as statistically significant.

## 3. Results

### 3.1. Power Spectral Density before and after Sonication

**Figure 6:**
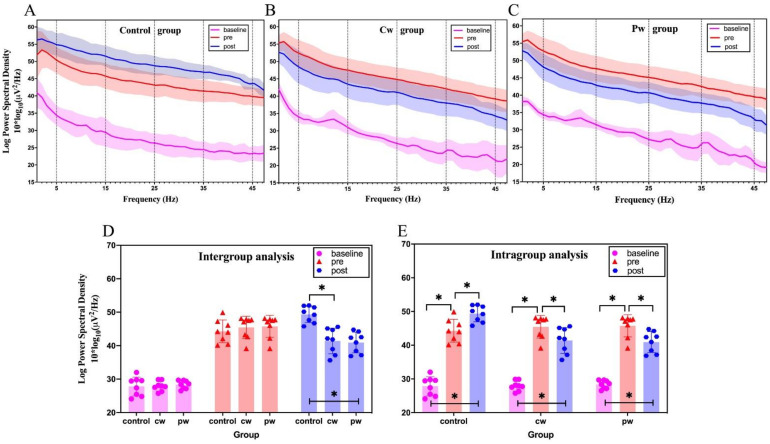
The raw EEG power spectral density (PSD). (**A**) Distribution of PSD in each frequency band in the control group. (**B**) Distribution of PSD in each frequency band in the cw group. (**C**) Distribution of PSD in each frequency band in the pw group. (**D**) The intergroup analysis of PSD in different stages. (**E**) The intragroup analysis of the three groups. * indicates significance (*p* < 0.05).

We performed study analysis on the preprocessed raw EEG of the three groups, calculated the raw EEG power spectral density (PSD), and compared the power spectral densities within and between groups. It can be seen from Figure 6 that after the injection of KA, the PSD in the three groups increased compared with the baseline stage in each frequency band. The intergroup analysis revealed no significant difference in the baseline stage and pre-stages (Figure 6D). After cw and pw stimulation, the PSD of the post-stage decreased, while the PSD of the control group continued to increase (Figure 6B,C). In the post-stage, intergroup analysis revealed no significant difference between the PSD of the cw group and pw group, while there were significant differences between the PSD of the two US-treated groups and the control group, as shown in Figure 6D. Figure 6E shows the intragroup analysis of the three groups. The PSD of the cw group and pw group significantly decreased after sonication, while the PSD of the control group significantly increased.

### 3.2. Phase Lag Index Changes before and after Sonication

**Figure 7:**
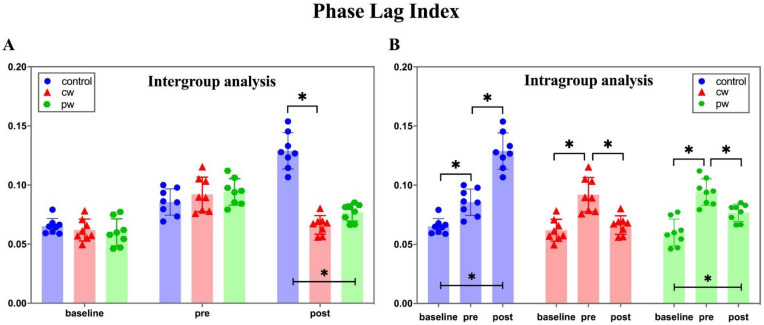
Mean phase lag index (PLI) for the broad-filtered band (0.1–48 Hz). (**A**) Intergroup analysis of the mean PLI at the different stages. (**B**) Intragroup analysis of the mean PLI in the three groups. * indicates significance (*p* < 0.05).

**Figure 8:**
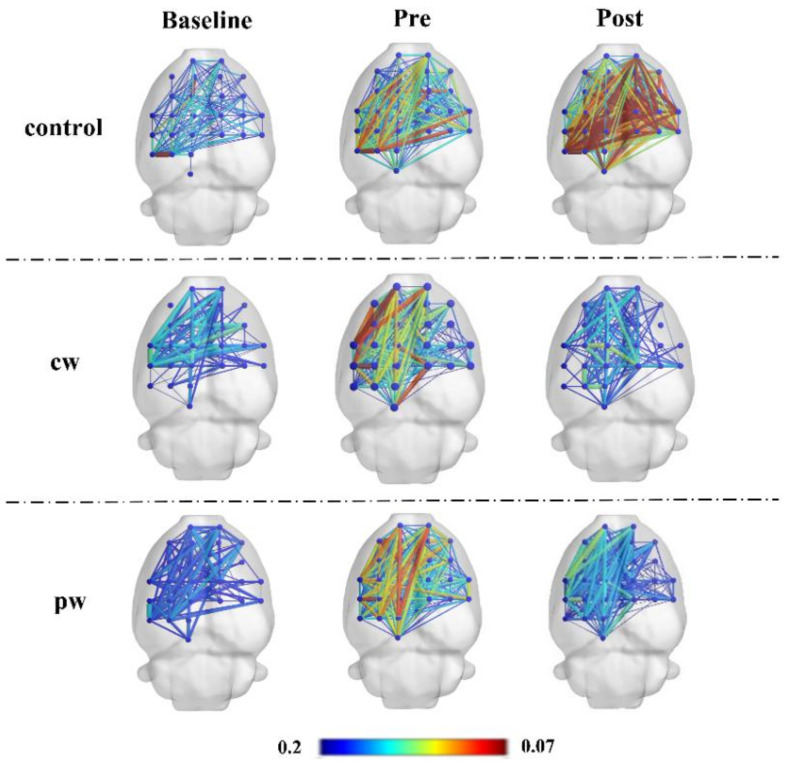
The differences in the strength of functional brain connections in three groups at different stages.

We calculated the PLI of each experimental animal in the broad-filtered band (0.1–48 Hz) and performed the statistical analysis. Intergroup analysis revealed no significant difference between the three groups in the baseline stage and the pre-stage before sonication, as shown in Figure 7A. After sonication in the cw group and pw group, PLI values of these two groups were significantly different from the control group in the post-stage (F(2, 21) = 77.12, *p* < 0.05), but there was no significant difference between the pw group and cw group. In the intragroup analysis, the PLI continued to rise after intraperitoneal injection of KA in the control group, with statistical significance between each stage (F(2, 21) = 63.18, *p* < 0.05). In the cw group (F(2, 21) = 17.56, *p* < 0.05) and pw group (F(2, 21) = 22.67, *p* < 0.05), PLI significantly decreased after sonication, as shown in Figure 7B. Figure 8 shows the changes in the brain functional connections of the broad-filtered band at three different stages. As shown in Figure 8, the strength of functional brain connections of the three groups had no obvious difference in the baseline stage and the pre-stage, while the connection strength of the cw group and pw group in the post-stage was obviously lower than that of the control group, which is consistent with the statistical analysis of the PLI.

### 3.3. Functional Connectivity Indicator Changes before and after Sonication

**Figure 9:**
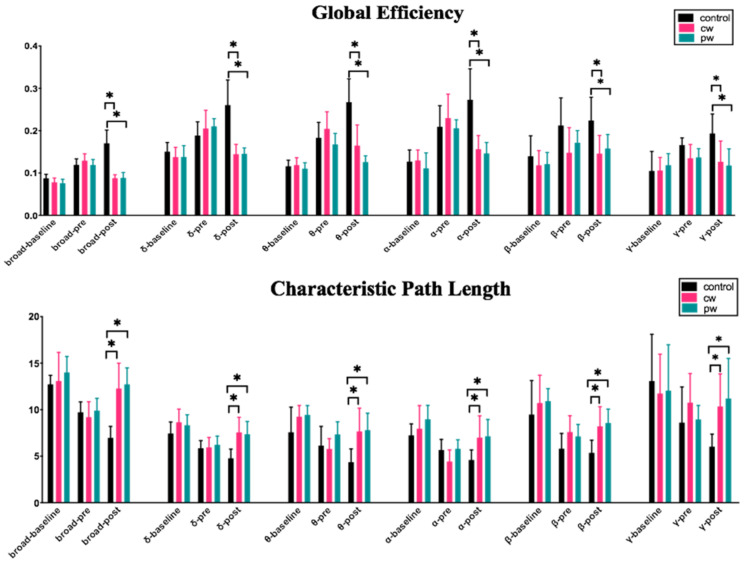

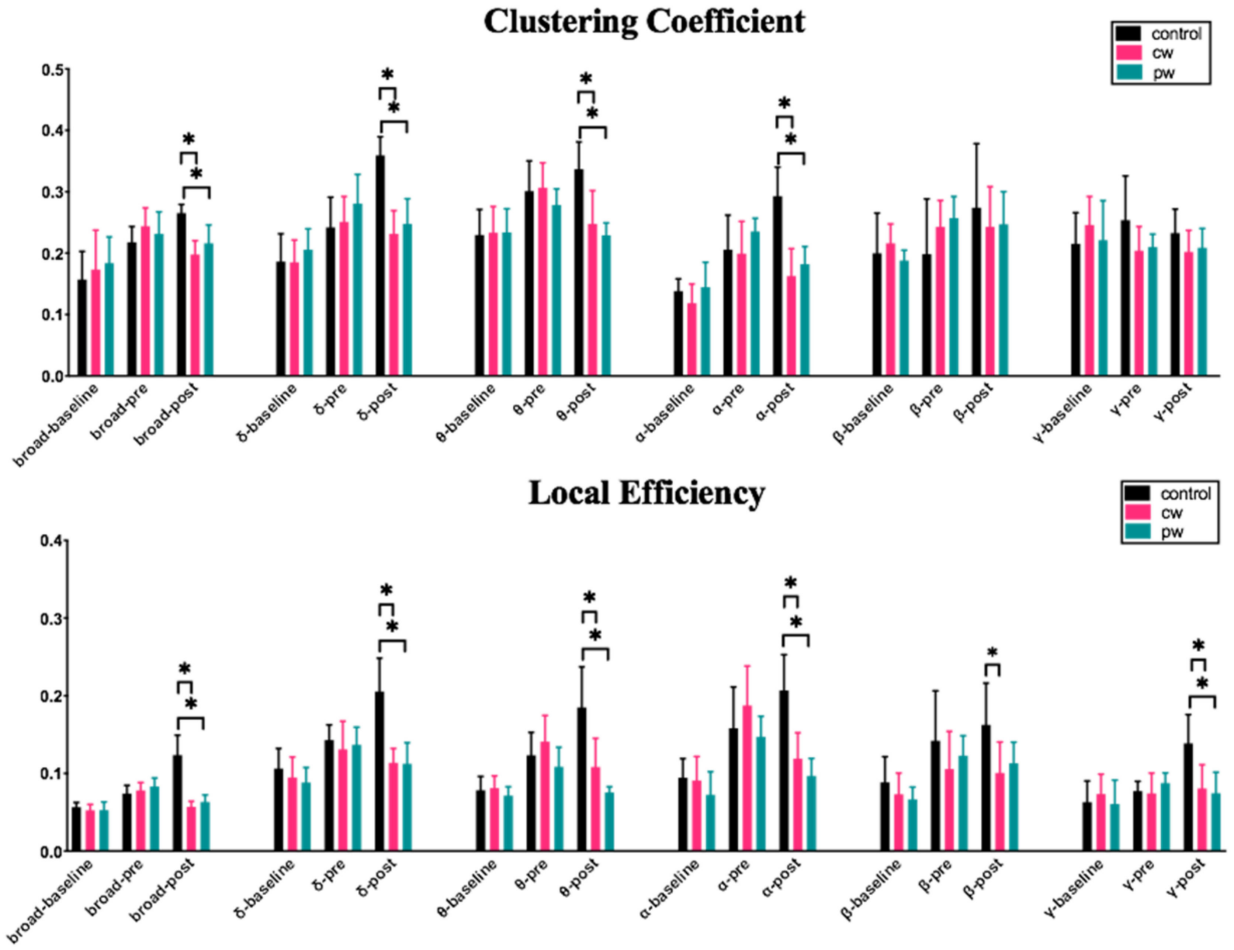
Intergroup analysis comparisons of brain functional connectivity indicators (L_p_, C_p_, E_g_, and E_loc_) for all filtered bands (delta 0.1–4 Hz, theta 4–8 Hz, alpha 8–13 Hz, beta 13–30 Hz, and gamma 30–48 Hz) and the broad-filtered EEG (0.1–48 Hz). * *p* < 0.05.

As shown in Figure 9, none of the baseline stage and pre-stage indicators in any of the three groups showed significant differences in each frequency band in the intergroup analysis, which proved that the three groups of rats had basically the same degree of seizure before sonication. L_p_ of all the bands in the control group was significantly lower than those in the cw group and pw group in the post-stage. C_p_ of the control group was significantly higher than those of the cw group and pw group in the broad, delta, theta, and alpha bands. Similar trends were observed in other frequency bands, but they were not significantly different. E_g_ and E_loc_ in the control group were significantly higher than those of the two US-treated rats in all the bands. None of the indicators of the post-stage in the cw group and pw group showed significant differences in each frequency band. Table 2 shows the intergroup analysis results of ANOVA among the three groups in the post-stage.

**Figure 10:**
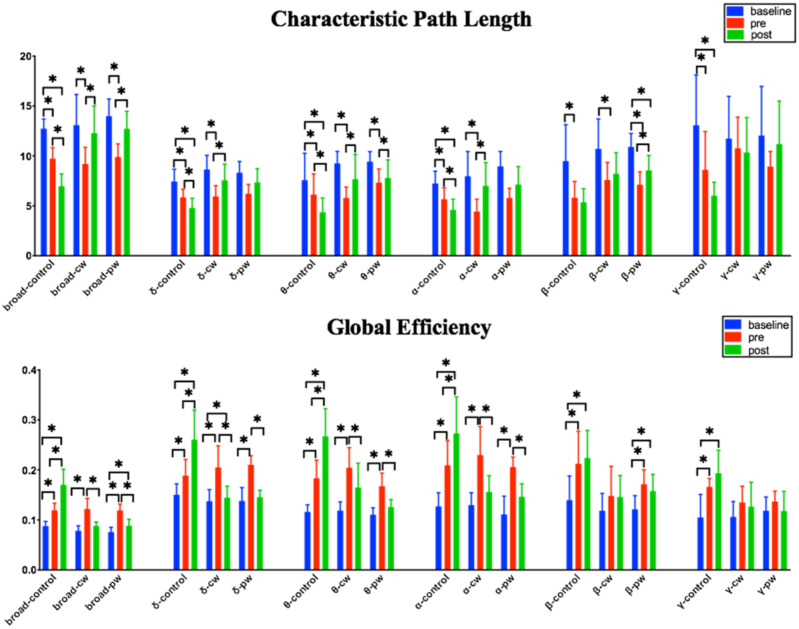

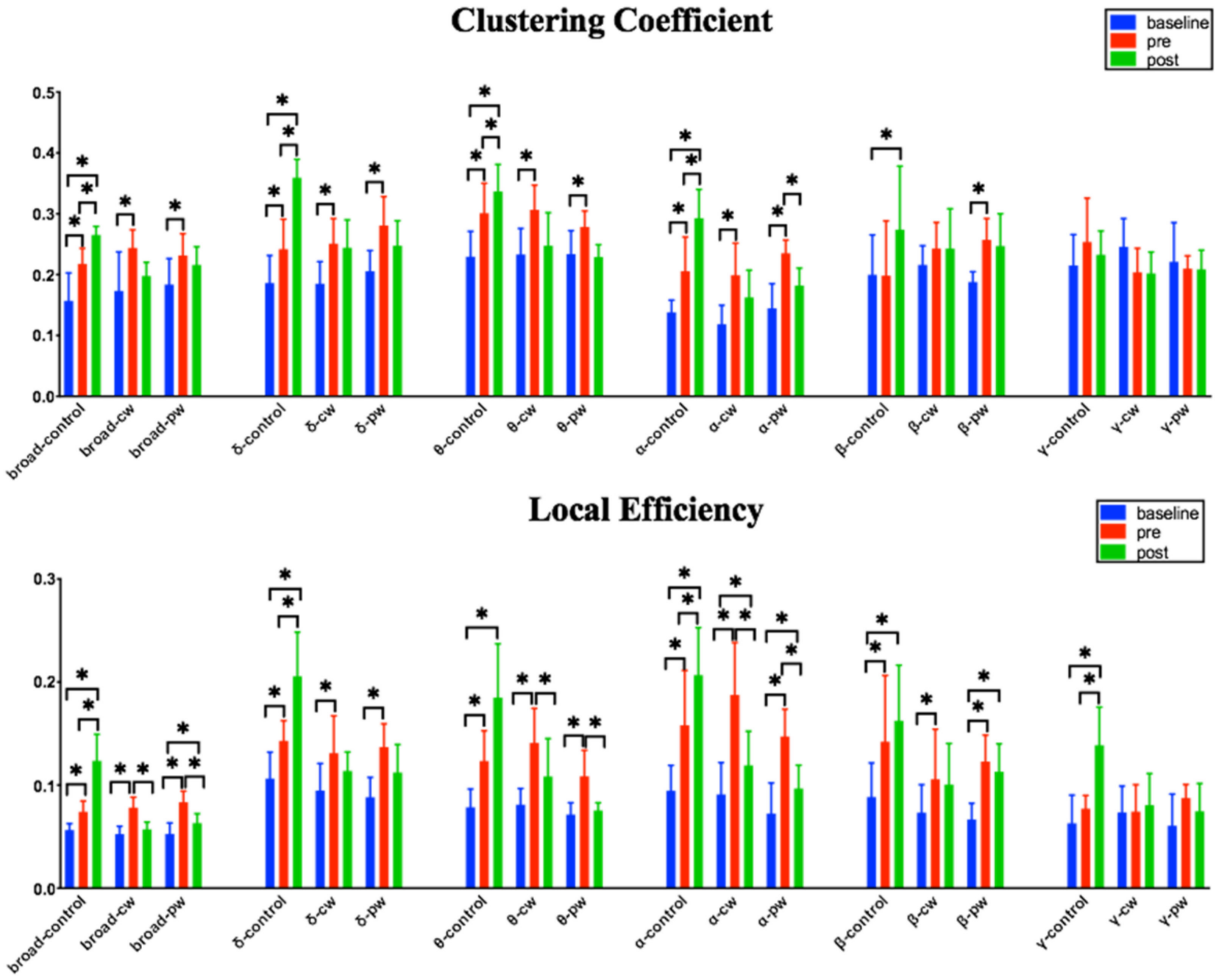
Intragroup analysis comparisons of brain functional connectivity indicators (L_p_, C_p_, E_g_, and E_loc_) for all filtered bands (delta 0.1–4 Hz, theta 4–8 Hz, alpha 8–13 Hz, beta 13–30 Hz, and gamma 30–48 Hz) and the broad-filtered EEG (0.1–48 Hz). * *p* < 0.05.

As shown in Figure 10, the intragroup analysis revealed that the four indicators had significant changes in all frequency bands after a seizure. In the intragroup analysis, the values of C_p_, E_p_, and E_loc_ in the control group showed a continuous increase in the three stages, as shown in Figure 10. For L_p_, the value of the control group was continuously decreasing. L_p_ showed an upward trend in the cw group and pw group after sonication in the frequency band below 30 Hz, while there was no significant difference in the gamma band. After cw and pw stimulation, C_p_ showed a tendency to decrease or remain unchanged in the post-stage. Although there was no significant difference, it was completely different from the significant increase in the control group. It is worth noting that the gamma band presented a completely different trend from the other bands. Global efficiency E_g_ showed a significant downward trend in the broad, delta, theta, and alpha bands after cw and pw stimulation, while there was no significant difference in the beta and gamma bands. The local efficiency E_loc_ significantly decreased in the broad, theta, and alpha bands after sonication, while in the control group, E_loc_ significantly increased during the normal course of a seizure.

### 3.4. Histological Examination of Brain Injuries

**Figure 11:**
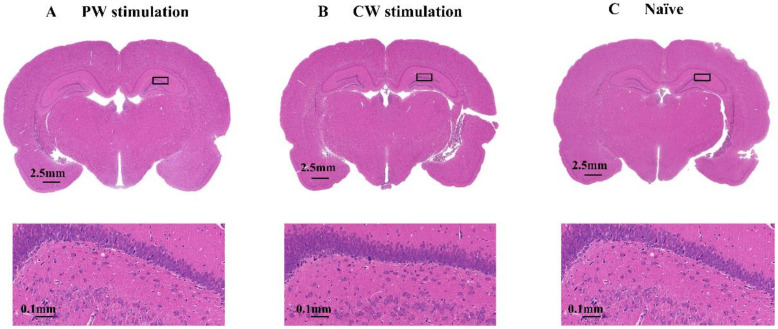
Histological examination of brain injury. (**A**) The eosin staining results of the pw stimulation. (**B**) The eosin staining results of the cw stimulation. (**C**) The eosin staining results of the naïve rat. A comparison of hematoxylin and eosin staining in brain sections obtained from US-treated and naïve rats was performed. These histological results were similar for naïve (US-) and US-treated brain sections (cw stimulation and pw stimulation). The large rectangles show a higher magnification of the hippocampus sections.

Sonication may induce neuron damage, thus mitigating KA-induced abnormal EEG bursts. To rule out this possibility, rats were treated with cw sonication and pw sonication or without sonication and were sacrificed 24 h later. No obvious adverse reaction was observed in rats over 24 h. Hippocampus sections of brains from US-treated and naïve animals were assessed for tissue damage (Figure 11). Low-magnification images revealed no obvious tissue damage in either the pw group or cw group. High-magnification H&E images showed no immune cell infiltration or microglia activation and expansion around the hippocampus area, which suggested that sonication did not cause damage or inflammation in rat brains.

## 4. Discussion

Our experimental results and analysis showed that both LICUS and LIPUS reduced the PLI value during epileptic seizures. In other words, the ultrasound neuromodulation inhibited the strength of the epileptic brain network connections. Several important brain network indicators were also changed: the clustering coefficient decreased, the characteristic path length increased, and the global efficiency and local efficiency decreased. In the control group without sonication, the strength of the brain network connections and the brain network indicators did not change, while an inverse trend was observed. To the best of our knowledge, this is the first report that investigated the power spectral density and brain network connections in epilepsy from different modes of low-frequency focused ultrasound-mediated attenuation of epilepsy. Our findings provide new ideas for the potential application of ultrasound neuromodulation.

The results of the power spectral density of the epileptic signals in the pre- and post-stages indicated that the two low-frequency focused ultrasound-mediated modes could attenuate epileptic seizures. The PLI values could be used to evaluate the strength of brain network connectivity. The higher the connection strength of the brain network, the higher the synchronization among the various areas of the brain. Our results showed that the PLI values in the post-stage of the cw group and pw group were significantly lower than those of the control group in the inter-group analysis, and they were also significantly decreased in the intragroup analysis relative to the pre-stage. The results of the control group showed that PLI significantly increased at all stages over time during normal seizures, thus indicating that in the early stage of epileptic seizures, the brain network connectivity of the brain network was enhanced, and the synchronization among the brain regions increased, which could greatly improve the efficiency of epileptic signal transmission in the whole brain. The ultrasound neuromodulation could reduce the connection strength of the brain network; however, the pulsed ultrasound stimulation and continuous ultrasound stimulation of the same frequency and intensity did not significantly differ. It seems that these two modes of ultrasound could inhibit the epileptic brain network, achieving similar results.

The local information transmission capability of the network can be measured and evaluated by the clustering coefficient, which the higher clustering coefficient stands for, the stronger local transmission capability, and correspondingly, the greater local efficiency of the network. In the cw group and pw group, the clustering coefficient and local efficiency significantly decreased after sonication, especially in the frequency band below 12 Hz, while in the control group, the clustering coefficient and local efficiency significantly increased over time in the course of a normal epileptic seizure. It can be seen that the trends of C_p_ and E_loc_ were mutually supported. Both continuous ultrasound stimulation and pulsed ultrasound stimulation could slow down the increase and even reduce the local transmission capacity of the epileptic brain network. As shown by the intergroup analysis, there were no significant differences between the results of the cw group and the pw group, which indicated that the effects of the two low-frequency focused ultrasound modes on the local transmission capability of the epileptic brain network were similar.

The characteristic path length evaluates the global information transmission capacity of networks. The stronger the global information transmission capacity of the network, the smaller the characteristic path length, and the greater the global efficiency of the network. In the control group, the characteristic path length significantly decreased, and the drop change was more significant in the frequency band below 12 Hz especially. However, in the cw group and pw group, the characteristic path length had upward trends, although there were no statistically significant differences in some frequency bands. The changing trend of the global efficiency was exactly the opposite of the changing trend of the characteristic path length. The increase in global efficiency and local efficiency was conducive to the transmission of epileptic signals in the whole brain and the local brain region and allowed for a high degree of synchronization among various brain regions. Low-frequency focused ultrasound neuromodulation could inhibit transmission of epileptic signals and impede the highly synchronized development among various brain regions, especially in the frequency band below 12 Hz. Moreover, our results proved that the transmission mode of ultrasound did not affect the inhibitory effect of ultrasound on acute epilepsy. Both continuous ultrasound and pulsed ultrasound could achieve the regulation of the epileptic brain network.

Gavrilov et al. reported that ultrasound stimulates the neural structures by applying mechanical force, which could cause alterations in membrane potential, thereby stimulating the nervous system [58]. In addition, it has been suggested that ultrasonic neuromodulation may affect the membrane’s fluidity, turbidity, and permeability [18,59]. Tyler et al. proposed that ultrasound could motivate mechanical waves in neuronal membranes, consequently depolarizing them adequately to activate voltage-gated ion channels and trigger action potentials [60]. At low intensities for short exposure times, the mechanisms underlying the effects of ultrasound on neuronal activity are thought to partially stem from the mechanical pressure effects of ultrasound on cellular membranes and ion channels without tissue heating [20,61,62,63,64]. It has been consistently reported that voltage-gated Na^+^ and Ca^2+^ channels can be activated by ultrasound sonication [21] and that several mechano-sensitive ion channels can be activated by ultrasound-mediated mechanical force, allowing cation entry [65,66,67] and causing alterations in membrane potential [68]. This is just one of many possible hypotheses on the mechanisms through which ultrasound regulates neuronal activity. Even if the exact mechanisms of action remain unknown, ultrasound neuromodulation represents a forceful new method in the field of neuroscience. The treatment of epilepsy is a representative application of ultrasound neuromodulation. Seizures are caused by abnormally excessive or synchronous neural activity in the brain, synaptic contact between neurons could potentially be destroyed by ultrasound waves [69], and synaptic contact between neurons could potentially be destroyed by ultrasound waves [70], where ultrasonic sonication might reduce the transmission efficiency of epileptic signals across the brain. KA mainly causes abnormal neuronal hyperexcitability and histopathological lesions in the bilateral hippocampus of the brain, similar to human temporal lobe epilepsy [71,72]. According to our experimental results, it can be hypothesized that ultrasound controls neural circuits and the central nervous system by affecting functional brain connections, especially the low-frequency band below 12 Hz, as the main frequency band of the hippocampus. This can also explain the significant changes in the indicators of the brain network connections before and after ultrasonic sonication.

While ultrasound neuromodulation has been gaining increasing attention, little is known on the range of acoustic parameters and different modes of ultrasound that elicit a response. Li et al. found that continuous and pulsed waves both decreased the intensity of the power spectrum at low frequencies (<10 Hz) and increased the interval between seizures, while the phase-amplitude coupling strength between slow (delta-, theta-, and alpha-frequency bands) and fast (gamma-frequency bands) neural oscillations was weakened [30]. King et al. found that continuous-wave stimuli were equally, if not more, effective than pulsed-wave stimuli were in eliciting responses [29]. Our experimental results showed no significantly different effects between the results of continuous-wave stimuli and pulsed-wave stimuli on the power spectral density of epilepsy signals or the indicators of the epileptic brain network, thus indicating that the two modes of ultrasound can both effectively regulate the epileptic brain network. In summary, these findings revealed that transcranial focused sonication provided a significant suppressive effect on the KA-induced epileptic brain network in rats. Despite the hypothalamus of rats being able to respond to ultrasound [73] and even induce audiogenic seizures [74,75], our observations are unlikely to be related to the rats’ auditory responsiveness to ultrasound. The reason is that the rodents’ audible range (approximately 30 to 70 kHz) of ultrasound frequencies was much smaller than the ultrasound frequency in this study (500 kHz) [73]. In general, the rodent species used in this study can handle ultrasound up to approximately 80 kHz [76].

Another thing we need pay attention to is that ultrasound treatment can potentially generate free radicals [77,78]. Although these free radicals are short-lived and extremely unstable, they can react easily with other biomolecules around and cause tissue damage or inflammation [79]. Yet, these free radicals are usually generated at high ultrasound frequencies associated with cavitation [80]. As the results of histological analysis showed (cf. Figure 11), the ultrasound applied in this research did not result in any unintentional biological damage to the targeted area of the brain. In this paper, we used hematoxylin and eosin (H&E) to evaluate potential hemorrhaging and microscopic tissue injuries, where only gross lesions could be detected, and quantitative immunohistochemical experiments were not performed. In future research, we will consider using Fluoro-Jade B (FJB) for staining to evaluate neuronal cell death in brain regions and anti-neuron-specific nuclear protein (NeuN) to evaluate neuronal cell survival. Brain sections can be stained against anti-glial fibrillary acidic protein (GFAP), a marker of astrocytes, which are absent in lesioned areas, in order to quantitatively assess changes in the hippocampus [48].

KA induces a status epilepticus (SE), the dynamics of which is pretty complex. Costa et al. investigated the relationship between epileptogenesis and lesion extent or dynamics [48]. Our experiment only explored the effect of ultrasound neuromodulation in the acute epilepsy model on the brain network, and it is still unknown how SE will be affected by the ultrasound neuromodulation. Costa et al. found that the onset of chronic epilepsy is modulated by SE dynamics, whereas brain damage is related to prolonged convulsions in SE. Therefore, in SE, whether ultrasound neuromodulation can further relieve convulsions and reduce the degree of brain damage remains to be further studied [48]. Several researchers have suggested that the possible existence in the central nervous system of modes of intercellular communication, other than classical synaptic transmission, hydrogen ions, temperature gradients, and pressure waves, may be regulators of wiring transmission and volume transmission in the intercellular communication in the central nervous systems [81]. The effects of pressure and temperature changes caused by ultrasound neuromodulation on the central nervous system should also be considered in future studies.

## 5. Conclusions

Our results suggest that ultrasound neuromodulation can effectively regulate epileptic brain connections and that the ultrasound-mediated attenuation of epilepsy is independent of modes of ultrasound. US-mediated region-specific functional neuromodulation is expected to become a powerful method for studying brain function and neurological diseases, while the effect of US on brain network connections may provide a new research direction for the mechanism of ultrasound neuromodulation.

In our future research, we plan to study the different effects of more ultrasound parameters on brain network connections and carefully select ultrasound parameters to formulate reasonable and detailed treatment guidelines. In addition, we will consider extending the experimental period and take necessary technical measures to monitor the behavior of animals.

## Figures and Tables

**Table 1:** Brief descriptions of complex network indicators in this paper.

Indicator	Character	Description
Characteristic path length	L_p_	The extent of the overall routing efficiency of a network
Clustering coefficient	C_p_	The extent of local clustering or cliquishness of a network
Global efficiency	E_g_	How efficiently information is propagated through the whole network
Local efficiency	E_loc_	How efficiently information is propagated to the direct neighbors of a node

**Table 2:** Intergroup comparisons of brain network.

Frequency	Lp	Cp	Eg	Eloc
**0.1–48 Hz**	F(2, 21) = 20.42; *p* < 0.0001	F(2, 21) = 18.38; *p* < 0.0001	F(2, 21) = 44.16; *p* < 0.0001	F(2, 21) = 40.87; *p* < 0.0001
**0.1–4 Hz**	F(2, 21) = 10.62; *p* = 0.0006	F(2, 21) = 28.71; *p* < 0.0001	F(2, 21) = 25.03; *p* < 0.0001	F(2, 21) = 23.49; *p* < 0.0001
**4–8 Hz**	F(2, 21) = 7.884; *p* = 0.0028	F(2, 21) = 14.89; *p* < 0.0001	F(2, 21) = 22.69; *p* < 0.0001	F(2, 21) = 18.34; *p* < 0.0001
**8–12 Hz**	F(2, 21) = 4.918; *p* = 0.0177	F(2, 21) = 23.37; *p* < 0.0001	F(2, 21) = 16.90; *p* < 0.0001	F(2, 21) = 21.71; *p* < 0.0001
**12–30 Hz**	F(2, 21) = 8.622; *p* = 0.0018	F(2, 21) = 0.189; *p* = 0.8290	F(2, 21) = 7.068; *p* = 0.0045	F(2, 21) = 4.933; *p* = 0.0175
**30–48 Hz**	F(2, 21) = 5.646; *p* = 0.0109	F(2, 21) = 1.670; *p* = 0.2122	F(2, 21) = 6.766; *p* = 0.0054	F(2, 21) = 9.921; *p* = 0.0009

## Data Availability

The datasets obtained during the current study are available from the corresponding author on reasonable request.
